# Lithium increases mitochondrial respiration in iPSC-derived neural precursor cells from lithium responders

**DOI:** 10.1038/s41380-021-01164-4

**Published:** 2021-06-01

**Authors:** Jordi Requena Osete, Ibrahim A. Akkouh, Denis Reis de Assis, Attila Szabo, Evgeniia Frei, Timothy Hughes, Olav B. Smeland, Nils Eiel Steen, Ole A. Andreassen, Srdjan Djurovic

**Affiliations:** 1 Department of Medical Genetics, Oslo University Hospital, Oslo, Norway; 2 NORMENT, Institute of Clinical Medicine, University of Oslo, and Division of Mental Health and Addiction, Oslo University Hospital, Oslo, Norway; 3 NORMENT, Department of Clinical Science, University of Bergen, Bergen, Norway

**Keywords:** Molecular biology, Bipolar disorder

## Abstract

Lithium (Li), valproate (VPA) and lamotrigine (LTG) are commonly used to treat bipolar disorder (BD). While their clinical efficacy is well established, the mechanisms of action at the molecular level are still incompletely understood. Here we investigated the molecular effects of Li, LTG and VPA treatment in induced pluripotent stem cell (iPSC)-derived neural precursor cells (NPCs) generated from 3 healthy controls (CTRL), 3 affective disorder Li responsive patients (Li-R) and 3 Li non-treated patients (Li-N) after 6 h and 1 week of exposure. Differential expression (DE) analysis after 6 h of treatment revealed a transcriptional signature that was associated with all three drugs and most significantly enriched for ribosome and oxidative phosphorylation (OXPHOS) pathways. In addition to the shared DE genes, we found that Li exposure was associated with 554 genes uniquely regulated in Li-R NPCs and enriched for spliceosome, OXPHOS and thermogenesis pathways. In-depth analysis of the treatment-associated transcripts uncovered a significant decrease in intron retention rate, suggesting that the beneficial influence of these drugs might partly be related to splicing. We examined the mitochondrial respiratory function of the NPCs by exploring the drugs’ effects on oxygen consumption rate (OCR) and glycolytic rate (ECAR). Li improved OCR levels only in Li-R NPCs by enhancing maximal respiration and reserve capacity, while VPA enhanced maximal respiration and reserve capacity in Li-N NPCs. Overall, our findings further support the involvement of mitochondrial functions in the molecular mechanisms of mood stabilizers and suggest novel mechanisms related to the spliceosome, which warrant further investigation.

## Introduction

Bipolar disorder (BD) is an affective disorder with a prevalence of 2–3% in the general population [1]. It is a debilitating illness characterized by recurring episodes of depression and mania or hypomania. Among the most widely prescribed and studied medications for treating BD is lithium (Li), which has a full response rate of about 30% [2]. Li prevents recurrence of both manic and depressive episodes, and is the first-line treatment for long-term maintenance phase [3]. Valproate (VPA) is a leading alternative mood stabilizer, being most effective against mania [4]. Lamotrigine (LTG) is also a commonly prescribed mood stabilizer, which shows a greater efficacy for preventing and ameliorating depressive episodes rather than mania [5]. In addition to BD, both Li [6] and VPA [7] may also have beneficial effect in major depressive disorder (MDD).

Multiple studies have investigated the molecular effects of mood stabilizers in neuronal cells, and several putative mechanisms of action have been suggested [8–10]. For example, Li has been found to selectively reverse an early hyperexcitable phenotype in young neurons derived from Li responders [10]. Furthermore, Li reduces dopamine and glutamate levels and increases inhibitory neurotransmitters-related signals (e.g., GABA) [8]. It also inhibits glycogen synthase kinase-3β activity [11], which has been linked to the drug’s anti-manic properties [9]. Similarly, VPA appears to act by reducing the activity of NMDA-glutamate receptors and by enhancing GABAergic neurotransmission [12]. In contrast, LTG does not seem to modify any of these targets, its main mode of action being the blockage of voltage-gated sodium channels [13]. Interestingly, long-term treatment with Li and VPA was found to protect against methamphetamine-induced mitochondrial dysfunction and toxicity in animal models [14] and enhance cell respiration rate in a neuroblastoma cell line, as determined by measurements of mitochondrial membrane potential and mitochondrial oxidation [15]. At the transcriptional level, the molecular effects of mood stabilizers, Li in particular, have been studied in different experimental models, including animal models [16], neuroblastoma cells [17], neurons [18] and peripheral lymphoblastoid cell lines derived from BD patients [19]. These studies have demonstrated widespread effects on diverse cellular signaling pathways related to apoptosis, immune functions, protein processing and response to endoplasmic reticulum stress [19].

However, little attention has been directed to the transcriptional and functional effects of mood stabilizers in neural precursor cells (NPCs), which seem to have been undervalued, despite the fact that they exhibit neuron-like mitochondrial and energy metabolism properties [20], are the major cell type in the subventricular zone of the lateral ventricle and subgranular zone of the hippocampal dentate gyrus and are essential for the generation of new neurons through neurogenesis [21]. For example, aberrant adult neurogenesis in the dentate gyrus has been implicated in MDD [22]. Interestingly, some studies have demonstrated that Li increases neurogenesis in the dentate gyrus of rodent hippocampus in addition to influencing the proliferation and differentiation of NPCs in hippocampus both in vitro and in vivo [23]. Hence, it has been suggested that the stimulation of endogenous NPCs may explain why Li is associated with thicker cortex and larger subcortical volumes in BD patients [24]. Furthermore, iPSC-derived NPCs have been shown to be appropriate models for the study of mitochondrial diseases [20], for phenotypic and transcriptomic characterization [25], neurotoxicity [26] and expression-based drug screening in psychiatric disorders [27]. Hence, in this study, we investigated the effects of Li, VPA and LTG in iPSC-derived NPCs generated from patients with affective disorder (BD and MDD), with a particular focus on transcriptional and mitochondrial mechanisms. Our findings unveil a noteworthy spliceosome boost triggered equally by all three drugs as well as a Li and VPA-induced improvement of mitochondrial respiration.

## Methods

### Patient samples

Skin biopsy donors were recruited through the Norwegian TOP (Thematically Organized Psychosis) study. Information about recruitment procedures, inclusion and exclusion criteria and clinical assessments for the TOP study as a whole have been described in detail elsewhere [28]. For reprogramming and NPC differentiation, fibroblasts were isolated from 3 healthy controls (CTRLs) and 6 patients with affective disorder that were selected based on clinical information. Supplementary Table 1 summarizes the clinical characteristics of the study participants. Patients experiencing no symptoms or only mild symptoms during Li treatment (patients #4-#6) were considered Li responders (Li-R), while the other patients (#1-#3) were considered non-treated (Li-N) (Supplementary Table1). The group of Li-responders included a partial responder (patient#4), as during some periods poor Li compliance was indicated. Skin biopsies were obtained under sterile conditions from the 9 donors. All participants have given written consent and the study was approved by the Norwegian Data Protection Agency and the Regional Ethics Committee of the South-Eastern Norway Regional Health Authority (REK grant: #2012/2204). The authors assert that all procedures contributing to this work comply with the ethical standards of relevant guidelines and regulations.

### IPSC derivation

Fibroblasts isolated from CTRL and patient donors were grown and expanded in vitro in DMEM 10% fetal bovine serum, 1% penicillin streptomycin and 1% Glutamax. Cells were then reprogrammed with Sendai virus, transduced with the CytoTune™-iPS 2.0 Sendai Reprogramming Kit (ThermoFisher) containing Oct4, Sox2, Klf4, and c-Myc reprogramming factors. Virus was washed after 24 h. At 7 days after transduction cells were plated on Vitronectin and medium was changed to Essential 8 Flex Medium (ThermoFisher). Passaging of iPSCs was done every 3–4 days or at a maximum of 80% confluency by using EDTA 0,5mM for 3–5 min at 37 °C. Cells were split in 1:3 ratio on Geltrex-coated plates in the presence of 10µM ROCK inhibitor Y-27632 (Miltenyi Biotec). Complete virus elimination was corroborated in iPSCs at passage 10 by RT-PCR with specific SeV genome detection primers (TaqMan Mr04269880-mr, ThermoFisher). Each iPSC line was subjected to rigorous quality control at the Norwegian Core Facility for Human Pluripotent Stem Cell Research Centre, including phenotyping, regular monitoring of morphology and pluripotency marker expressions.

### IPSCs differentiation to NPCs

IPSCs from CTRLs and patients were differentiated into neural precursor cells (NPCs) following a previously published protocol [29] with some modifications. First, iPSCs on Geltrex-coated plates at a 15–20% density were differentiated to neuroepithelium with rosette formation in A-DMEM/F12, 1% penicillin streptomycin, 1% Glutamax and 1% N2 with the addition of SB431542, LDN193189 and XAV-939 (dual SMAD inhibition and Wnt inhibition). At day 7, rosette-like structures were isolated with Rosette Selection Reagent (Stemcell technologies) and 9x10^5^cells/well were plated on laminin coated plates. NPCs were then cultured in A-DMEM/F12, 1% P/S, 1% Glutamax, 1% N2 and 0,4% B27 supplemented with FGF2 at 2,5ng/ml changing the medium every other day during 5–6 days. NPCs were then passaged and cultured in A-DMEM/F12, 1% P/S, 1% Glutamax, 1% N2, 0,1% B27 in hypoxic conditions (3% O_2_) on laminin coated 6-well plates. Basal medium was equilibrated in the incubator O/N and FGF2 was added fresh daily to the cells to a final concentration of 10ng/ml. Cells were passaged with accutase (Sigma) every 4–5 days.

### Immunofluorescence

IPSCs and NPCs grown in chamber slides (ThermoFisher, #177437) were fixed with 4% paraformaldehyde in PBS for 20 min, permeabilized with 0,2% Triton X-100 in PBS and blocked in 6% donkey serum for 1hour. Then, cells were incubated with primary and secondary antibodies. Primary antibodies used were: anti-Nestin (mouse, 1:1000, Stem Cell Technologies, 60091.1); anti-Sox1 (rabbit, 1:1000, Stem Cell Technologies, 60095); anti-Sox2 (rabbit, 1:200, Stemgent, 09–0024); anti-Oct4 (mouse, 1:200, Stem Cell Technologies, 60093.1) and anti-Nanog (rabbit, 1:200, Stemgent, 09–0020). Secondary antibodies (Alexa Fluors, ThermoFisher) were anti-rabbit Alexa 488 (1:200, Invitrogen, A32790) and anti-mouse Alexa 594 (1:200, Invitrogen, A21203). Nuclei were stained with 4′,6-diamidino-2-phenylindole (DAPI, Roche, 10236276001) and then cover glasses (VWR, ECN631-1575) were mounted on top with Fluoromount-G (Invitrogen, #00-4958-02). Images were acquired using a Zeiss LSM 700 confocal microscope and were processed using Fiji software. Fields based on uniform DAPI staining were selected. 300 cells were analyzed for each NPC marker from all lines.

### Real-time quantitative PCR

Cells were lysed directly in their wells by adding lysis buffer. Total RNA was extracted with the RNeasy Plus Mini kit (Qiagen, #74136) following the manufacturer’s instructions. Reverse transcription of 0.5 μg total RNA to cDNA was performed with the High-Capacity cDNA Reverse Transcription Kit (Applied Biosystems, #4368814). The cDNA was then used for real-time quantitative PCR with Power SYBR Green Master Mix following the manufacturer’s instructions (Applied Biosystems). All reactions were carried out on a QuantStudio 12K Flex Real-Time PCR System (Applied Biosystems). Relative mRNA expression was calculated as ratios normalized against GAPDH by using the 2^−ΔΔCt^ method. Primers used for iPSCs and NPCs characterization are specified in Supplementary Table 2.

### Drug administration

IPSC-derived neural precursor cells from all 9 donors were expanded until passage 4–5 and then treated with 1 mM Li (Sigma, #4408), 30 µM VPA (Sigma, P4543), 25 µM LTG (Tocris, #1611) (all three diluted in DMSO) or 0.1% DMSO alone (vehicle control). In all cases, the clinically recommended therapeutic serum concentrations for the drugs were used. For LTG and VPA, the concentrations in vitro were reduced compared to the recommended in vivo concentrations, because when treating patients it is necessary to increase the concentration to compensate for the drug loss due to plasma protein binding, estimated to be 50 and 90%, respectively. We treated the NPCs from all donors with all three mood stabilizers, regardless of their prescribed treatments, to identify shared mechanisms of action using cells with the same genetic background. Treatment duration was 6 h and 1 week, with medium and drug refreshment on a daily basis for the 1 week condition.

### RNA extraction and sequencing

Total RNA was extracted from all 72 samples (DMSO, Li, VPA, and LTG treatment for each of the 9 donors at two time points) with the RNeasy Plus Mini Kit (Qiagen). Approximately 1 million cells were used as input. RNA yield was quantified with a NanoDrop 8000 Spectrophotometer (NanoDrop Technologies) and RNA integrity was assessed with Bioanalyzer 2100 RNA 6000 Nano Kit (Agilent Technologies). All samples had an RNA Integrity Number (RIN) above 8.5. Library preparation and paired-end RNA-sequencing were carried out at the Norwegian High-Throughput Sequencing Centre (www.sequencing.uio.no). Briefly, libraries were prepared with the TruSeq Stranded mRNA kit from Illumina which involves Poly-A purification to capture coding as well as several non-coding RNAs. The prepared samples were then sequenced on an Illumina NovaSeq SP platform at an average depth of 50 million reads per sample using a read length of 100 base pairs and an insert size of 350 base pairs.

### Data processing

Raw sequencing reads were quality assessed with FastQC (Babraham Institute). To pass the initial QC check, the average Phred score of each base position across all reads had to be at least 30. Reads were further processed by cutting individual low-quality bases and removing adapter and other Illumina-specific sequences with Trimmomatic V0.32 using default parameters [30]. HISAT2 [31] was then used to first build a transcriptome index based on ENSEMBL annotations, and then to map the trimmed reads to the human GRCh38 reference genome. To quantify gene expression levels, mapped reads were summarized at the gene level using featureCounts [32] guided by ENSEMBL annotations.

### Cell type deconvolution and differential expression (DE) analysis

Bioinformatical estimation of cell type abundances (deconvolution) was carried out with CIBERSORTx [33] using the web-interface (https://cibersortx.stanford.edu/) with default parameters, selecting 500 permutations for statistical testing. Expression signatures for 8 relevant cell types from Burke et al. [34]. were used as reference: iPSCs, NPCs, fetal replicating neurons, fetal quiescent neurons, oligodendrocyte progenitor cells (OPCs), adult neurons, astrocytes, and oligodendrocytes. Before conducting the DE analyses, genes with very low to zero expression were removed by filtering out any gene with ≤0.5 counts per million (CPM) in 3 or more samples (the smallest group size). Since two of the patient lines (#5 and #6) had a partial chromosomal loss on chromosome 6 encompassing ~7000 kb, all genes within this deleted region were also excluded. For DE analyses, limma-voom [35] was used. Since a paired design was used, all variables were perfectly matched across groups and no covariates were included. For the DE analyses, age and NPC abundance were adjusted for. In all analyses, a DE gene was considered significant if the FDR was <0.10. Pathway analyses of significant DE gene sets were conducted with the over-representation analysis tool clusterProfiler [36] using the enrichKEGG function after removing pathways related to human diseases. A pathway was considered significantly enriched if the FDR was <0.10.

### Enrichment analysis in human brain regions

To test whether the treatment-specific DE genes were enriched in any specific brain region, ABAEnrichment [37] was used. This tool uses two human brain expression datasets (adult and developmental) provided by the Allen Brain Atlas [38, 39] and assigns to each anatomical structure the genes that are expressed in that structure by applying an expression threshold. It then performs ontology gene set enrichment analyses to identify significant overlaps between a custom-provided list of input genes and the genes that are expressed in a specific brain area using either a hypergeometric test or a Wilcoxon-rank sum test. Three cutoff quantiles (0.5, 0.7, 0.9) were used to annotate genes to brain regions, and the Wilcoxon-rank sum test and the “adult” dataset were selected for the enrichment analyses. By default, all enrichment analyses were adjusted for multiple testing using the family-wise error rate (FWER) approach. Enriched brain areas were visualized with the R package cerebroViz [40].

### Intron retention rate (IRR) quantification

To detect retained introns and estimate the IRR, we used IRFinder [41], which implements an end-to-end analysis of intron retention from RNA-sequencing data. The BAM files generated after mapping of sequencing reads with HISAT2 were used as input. Differential intron expression analysis was performed with the DESeq2 package [42] and the IRR was defined as the ratio between intronic reads and the sum of intronic and normally spliced reads. A change in IRR between DMSO and drug-treated samples (Li, VPA, LTG) was considered significant if the magnitude of the change was no less than 20% and the FDR < 0.05.

### KaryoStat assay

KaryoStat GeneChip array (ThermoFisher) was used for karyotyping of iPSCs at passages 15–20. It allows for digital visualization of chromosome aberrations with a resolution similar to g-banding karyotyping. The size of structural aberration that can be detected is >2 Mb for chromosomal gains and >1 Mb for chromosomal losses. The array covers all 36,000 RefSeq genes, including 14,000 OMIM® targets with an optimized low-resolution DNA copy number analysis. The assay enables the detection of aneuploidies, submicroscopic aberrations, and mosaic events. Genomic DNA (gDNA) was extracted using the Genomic DNA Purification Kit (Catalog #: K0512) and quantified using the Qubit dsDNA BR Assay Kit (Catalog #: Q32850). For GeneChip preparation a total of 250 ng gDNA was processed according to manufacturer’s instructions. Briefly, Nsp I digested gDNA was ligated, PCR amplified, fragmented, labeled with biotin and hybridized onto a GeneChip array overnight. Chips were washed and stained using GeneChip Fluidics Station 450 and scanned using GeneChip Scanner 3000 7 G. The raw data was processed using Chromosome Analysis Suite 3.2 with NetAffx na33.1 (UCSC GRCh37/hg19). Output data were interpreted with the UCSC Genome Browser (https://genome.ucsc.edu/; GRCh37/hg19 assembly).

### Mitophagy assay

Mitophagy levels were analyzed in all 9 lines after 6 h and 1 week of 1 mM Li stimulation using the Mitophagy detection kit (Dojindo Molecular Technologies, #MD01-10). Briefly, Mitophagy Dye was added to the cells at 100 nmol/l and incubated for 30 min at 37 °C. The Dye accumulates in intact mitochondria and is immobilized through chemical bonds. During mitophagic damage induction, the injured mitochondria fuses to lysosomes and then Mitophagy Dye emits a high fluorescence intensity [43]. Cells were analyzed by flow cytometry in a Gallios Flow Cytometer 10 colors, 4 lasers from Beckman Coulter. Data was analyzed with Gallios flow cytometry software.

### Bioenergetic assessment

Cellular bioenergetics assessment of cells was performed using a Seahorse XFe24 extracellular flux analyzer (Agilent Seahorse XF Analyzers), as described previously [20]. Briefly, 50,000 cells were plated into each Geltrex-coated well of the XFe24-well plates and incubated at 37 °C overnight with 5% CO_2_. Then, growth medium was replaced with unbuffered media and cells were incubated for 60 min at 37 °C in a non-CO_2_ incubator to allow the media to reach equilibrium of temperature and pH before starting recording data. Both mitochondrial respiration (OCR or oxygen consumption rate) and anaerobic glycolysis (ECAR or extracellular acidification rate) measurements were recorded simultaneously. Baseline levels were first recorded and then 4 different chemical inhibitors (all at 1 μM and from Sigma) were added to test mitochondrial respiration function. All experiments were performed in technical quadruplicates. First, oligomycin, a complex V blocker, was added to inhibit oxidative phosphorylation (OXPHOS) and test respiration coupling to ATP synthesis. Carbonyl cyanide-p-trifluoromethoxyphenylhydrazone (CCCP), a protonophore uncoupling agent, was added to decrease the mitochondrial membrane potential (MMP), thus increasing respiration rate and enabling the quantification of the maximal respiration under maximal mitochondrial uncoupling. The last compounds to be added were rotenone, a blocker of the complex I, and antimycin A, a blocker of the complex III, which cause complete inhibition of mitochondrial respiration, therefore allowing us to examine the non-respiratory oxygen consumption. Values were normalized to the total protein content in each well of the plate using the BCA protein assay kit (ThermoFisher, #23225).

### Statistics

Data are presented as mean ± SD for the iPSC and NPC characterization by RT-PCR and mitophagy assay results; and as mean ± SEM for the Seahorse results. In vitro mitophagy assay results represent mean values of duplicate measurements and Seahorse values represent the mean from quadruplicate measurements. Comparisons between groups were done by analysis of variance (ANOVA) between groups for the initial IRR analysis (F statistic and *p* value are shown in the figures). For the Seahorse parameters, ANOVA was used to compare the mean values of more than two groups, and two-tailed unpaired *t*-test was used to compare differences in drug treatment.

## Results

### Differentiation of iPSCs to NPCs

**Fig. 1:**
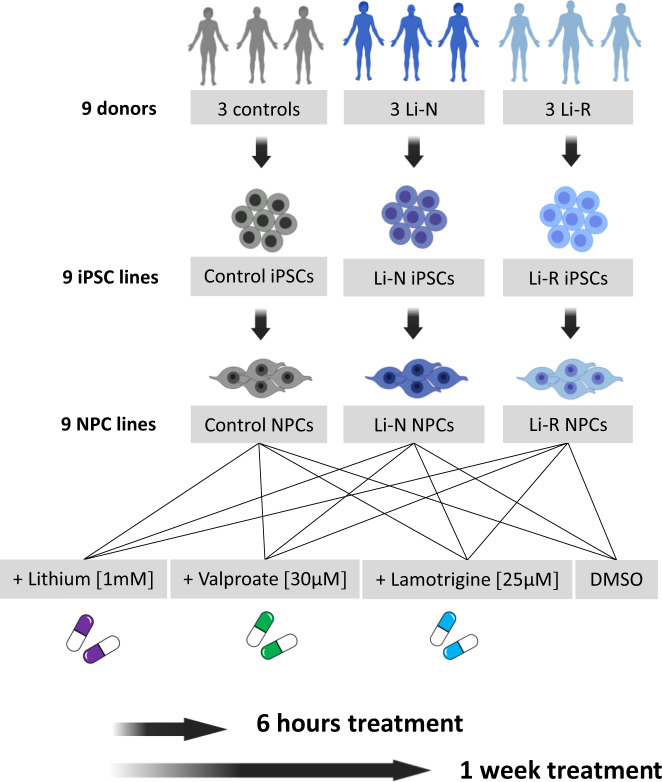
Schematic representation of the experimental design for the drug treatment of NPCs. Fibroblasts isolated from 3 CTRLs and 6 affective disorder patients, including 3 Li-N and 3 Li-R, were first reprogrammed into iPSCs and then differentiated into NPCs. All 9 lines were incubated with: 1 mM lithium (Li), 30 µM valproate (VPA), 25 µM lamotrigine (LTG), and DMSO as vehicle control, using the medically recommended therapeutic concentrations. The short-term effect of drug exposure (6 h and 1 week) were assessed through transcriptional profiling and functional analyses. NPCs neural precursor cells, IPSC induced pluripotent stem cells, CTRL control, Li-R lithium responders, Li-N lithium non-treated, DMSO dimethyl sulfoxide.

Fibroblasts from skin biopsies were obtained from 3 healthy controls (CTRL) and 6 affective disorder patients, of whom 3 were Li responders (Li-R) and 3 were Li non-treated (Li-N). The fibroblasts were reprogrammed to iPSCs using Sendai virus, and differentiated to NPCs following a previously published protocol [29]. NPC samples were subsequently treated with Li, VPA, and LTG, and RNA-seq was used for gene expression profiling (Fig. 1). All three groups were matched with respect to gender (2 females and 1 male per group). Details about donor age, gender, and diagnostic subtype are provided in Supplementary Table 1.

**Fig. 2:**
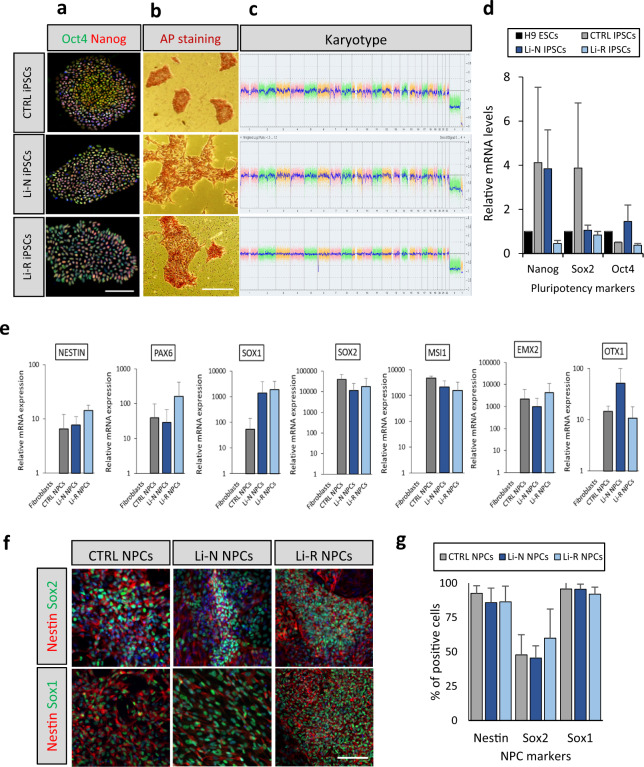
Characterization of iPSCs and NPCs. **a** Representative images of iPSC immunofluorescence staining for one line from each group (CTRL, Li-N and Li-R). Nuclear Oct4 and Nanog pluripotency markers are shown in the merged image. Cell nuclei were stained with DAPI. Scale bars: 100 µm. **b** Key pluripotency marker alkaline phosphatase (AP) staining positively in iPSC colonies from one line representative of each group used in the study. Scale bars: 100 µm. **c** Karyotype for one representative line from each group assessed by KaryoStat™ analysis. Somatic and sex chromosomes are displayed together. The y-axis shows the log2 ratios depicting the microarray probe’s signal intensities. A CN value of 2 represents a normal copy number state. Chromosomal gains are represented by a value of 3, while chromosomal losses are represented by a value of 1. Pink, green and yellow colors indicate each individual chromosome probe´s raw signal. The blue line represents the normalized probe signal used to identify copy number aberrations. The same ~7000 kb partial chromosomal loss was detected in 2 patient lines (#5 and #6) on chromosome 6 at position q24.3 (see also Supplementary Fig. 1c). **d** Real-time PCR analysis of the pluripotency marker genes *OCT4*, *SOX2* and *NANOG* in CTRL and patient-derived iPSCs compared to H9 embryonic stem cells (H9 ESCs) expression levels. Data is presented as mean ± SD. **e** Real-time PCR characterization of NPC marker genes *NESTIN*, *PAX6*, *SOX1*, *SOX2*, *MSI1*, *EMX2*, and *OTX1*. Values are given for mRNA expression levels relative to fibroblasts. Data is presented as mean ± SD. **f** NPC immunofluorescence staining for Nestin, Sox1 and Sox2. Scale bars: 100 µm. **g** Percentage of cells staining positive for Nestin, Sox2 and Sox1 by counting 300 cells from the immunofluorescence analysis of each line. No differences were found between groups.

The iPSCs were characterized by immunofluorescence, RT-PCR, and alkaline phosphatase staining for assessment of pluripotency marker expression, as well as karyotyping for genomic integrity testing. All iPSC lines expressed the pluripotency markers Oct4, Sox2, Nanog and AP (Fig. 2a–d and Supplementary Fig. 1a, b). Karyotyping revealed a near-identical partial loss in two of the patient lines (#5 and #6) at the 6q24.3 locus (Fig. 2c and Supplementary Fig. 1c). Deletions within the 6q24-25 region have been previously linked to both BD and suicidal behavior [44], and this region encompasses several genes which are implicated in BD, such as *SYNE1 or ZDHHC14* [45]. The size of the detected region was 6913 kb and 6992 kb in the two lines, respectively, and included ~30 genes with detectable expression levels in the NPCs (Supplementary Fig. 2a). Consequently, these genes were excluded before conducting the DE analyses.

Next, one iPSC clone from each donor was differentiated into NPCs by dual SMAD and Wnt inhibition and characterized at both mRNA and protein levels (Fig. 2e–g). All NPC cultures displayed high mRNA expression of the essential markers *NESTIN*, *PAX6*, *SOX1*, and *SOX2* (Fig. 2e). CTRL NPCs exhibited moderate expression of *SOX1* mRNA compared to patient NPC lines, while also showing slightly higher *SOX2* levels, but no statistically significant differences were detected between CTRL and patients (Fig. 2e). Donor NPCs also displayed high levels of *EMX2* and *MSI1* (Fig. 2e), which are expressed predominantly in proliferating multipotent neural precursor cells in the adult brain in regions such as the dentate gyrus [46, 47]. In addition, cells showed low expression of *OTX1* (Fig. 2e, which regulates neural progenitor cells in the developing cortex [48]. At the protein level, characteristic NPC markers were expressed by all NPC lines, and their phenotype was further validated by immunofluorescence of NESTIN, SOX2, and SOX1 at passage 3–4 after rosette selection, with no observable morphological differences between patient and CTRL cells (Fig. 2f, g and Supplementary Fig. 2b), indicating a successful generation of iPSC-derived NPCs.

### Transcriptomic characterization of NPC cultures and effect of mood stabilizers

Having established that the iPSC-generated cells display typical NPC markers, the short-term (6 h and 1 week) transcriptional effects of Li, VPA, and LTG treatment on global gene expression were assessed. The relatively short treatment durations were chosen, as longer drug exposure would require additional passaging of the NPCs, which may change the cellular phenotype.

**Fig. 3:**
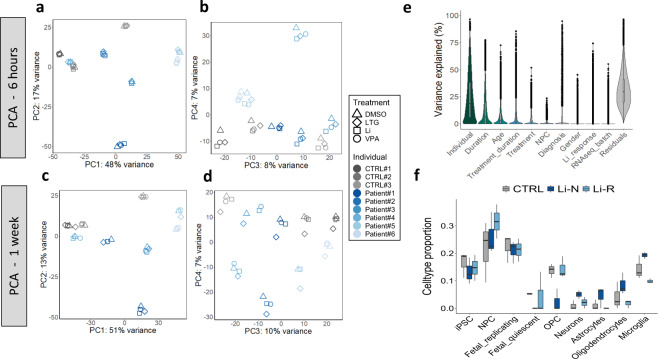
Transcriptomic characterization of NPC cultures. **a**–**d** Principal component analysis (PCA) plots of all samples based on the 1000 most variant genes. Most variation in gene expression is explained by donor effects. Within each cluster, treatment duration per se, i.e., regardless of the type of treatment, has a big effect on gene expression, underscoring the importance of including separate control samples (DMSO) for both duration categories. **e** Variance partition plot showing the proportion of gene expression variance attributed to different sources. Residuals constitute additional, unknown sources of variation not accounted for. **f** Cell type proportions estimated by computational deconvolution. NPCs and fetal replicating neurons were the cell populations with largest fractions, but other cell types are also present in the cultures. No significant differences between groups were found for any cell type. Fetal_replicating: Replicating neuronal progenitors (from fetal brain tissue, 16–18 weeks post-conception). Fetal_quiescent: Quiescent newly born neurons (from fetal brain tissue, 16–18 weeks post-conception). OPC oligodendrocytes precursor cells.

Principal component analysis (PCA) showed clear separation on donor when clustering across the first two principal components (PCs), explaining most of the variation in gene expression (Fig. 3a, c; Supplementary Fig. 2c). The importance of donor effects in iPSC studies is well established [49]. Within each cluster, regardless of the type of treatment, treatment duration had a large impact on gene expression (Fig. 3e). This observation highlights the need to include separate control samples (DMSO) for each duration (Supplementary Fig. 2c). A weak tendency of clustering on diagnosis (CTRL vs. patients) was seen when clustering across PCs 3 and 4, accounting for a smaller portion of the total variation (Fig. 3b, d). These findings were generally confirmed by variance partition analysis, which also attributed relatively small effects to RNA-seq batch, Li response status, and gender (Fig. 3e). Importantly, age had the third largest contribution to gene expression variation, which is in line with previous publications showing age as an influential factor in iPSC models [50], underscoring the necessity of adjusting for age differences in DE analyses.

Since differences in cell type proportions are another major source of gene expression variance [49], we used computational deconvolution to estimate the abundances of 8 relevant brain cell populations. As expected, NPCs constituted the largest fraction of cells in the cultures, but other cell types were also present (Fig. 3f). In particular, a relatively high proportion of fetal replicating neurons were detected, suggesting transcriptional similarities between our cell cultures and early progenitor stages (16–18 weeks post-conception) of fetal brain tissue. Although none of the differences in cell type composition between groups were statistically significant, we adjusted for NPC proportion in the DE analyses due to a higher median estimate in Li-R samples compared to Li-N and CTRLs.

### Genes modulated by mood stabilizers are enriched in cerebellar brain regions and involved in ribosome and oxidative phosphorylation pathways

To investigate how common mood stabilizers modulate gene expression, and how this modulation is affected by disease and Li response status, three sets of DE analyses were carried out: 1) The first set consisted of DE analyses between DMSO and mood stabilizer treatment (Li, VPA, LTG) combining both CTRL and patient samples, with the aim of identifying genes whose expression profile is associated with the drug treatment per se (treatment-associated genes). 2) The second set consisted of DE analyses between DMSO and drug treatment in patients only, to identify DE genes that are specifically regulated in patients upon drug exposure (treatment-patient-associated genes). 3) The third set consisted of DE analyses between Li treatment and Li response status (Li-N vs. Li-R), to identify genes that are differentially expressed in Li-R but not in Li-N patients after Li treatment (Li-response-associated genes).

**Fig. 4:**
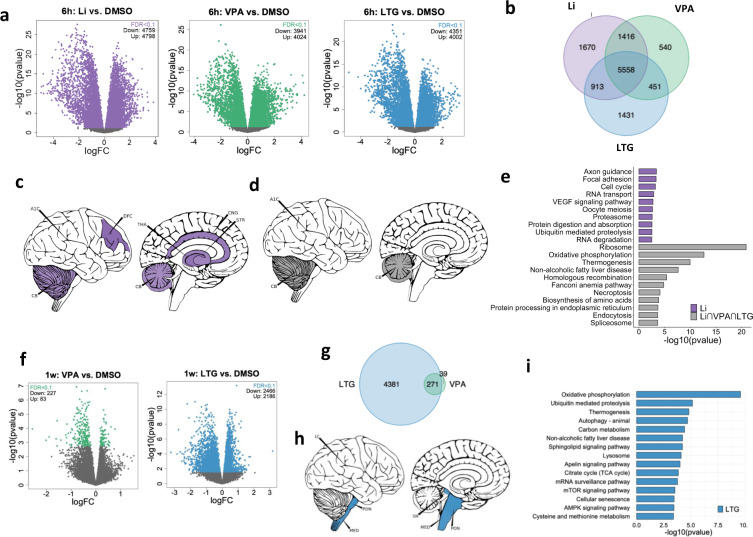
Treatment-specific DE genes and downstream analyses after 6 h and 1 week of exposure. **a** Volcano plots of DE genes for each treatment (Li, VPA and LTG) vs. DMSO after 6 h of exposure. **b** Venn diagram of DE genes after 6 h of exposure. The majority of DE genes were shared between all three treatments. **c** Enrichment of 6 h Li-specific genes in human brain structures. **d** Enrichment of 6 h shared DE genes in human brain structures. **e** Pathway (KEGG) analysis of DE genes (1670) that were unique to Li treatment (purple) and DE genes (5558) that were shared (gray) between all three treatments. The DE genes uniquely associated with VPA (540) and LTG (1431) were not enriched for any pathways. **f** Volcano plots of DE genes associated with VPA and LTG treatment vs. DMSO after 1 week of exposure. Li treatment for 1 week did not result in any significant DE gene. **g** Venn diagram of DE genes associated with 1 week LTG and VPA treatments. **h** Enrichment of 1 week LTG genes in brain regions. **i** Pathway (KEGG) analysis of unique LTG genes. No significantly enriched pathway was identified for unique VPA genes. VPA valproate, LTG lamotrigine, DMSO dimethyl sulfoxide, KEGG the Kyoto Encyclopedia of Genes and Genomes, CB cerebellum, MED medulla oblongata, PON pons, SN substantia nigra, CNG anterior cingulate cortex, DFC dorsal frontal cortex, THA thalamus.

After 6 h of treatment with Li, VPA, and LTG, a substantial effect on gene expression was found, with 9557 (54%), 7965 (45%), 8353 (47%) DE genes being significantly associated (FDR < 0.1) with each of the drugs, respectively. In all cases, approximately half of the treatment-associated DE genes were up-regulated and the other half was down-regulated (Fig. 4a). Moreover, 5558 genes were associated with all three drugs (Li∩VPA∩LTG), indicating a large overlap in their gene expression signatures (Fig. 4b). To examine whether the identified DE genes are over-represented in any specific brain regions, we performed human brain enrichment analysis using the top 1000 DE genes as input. Genes uniquely associated with Li were significantly enriched (FDR < 0.05) in several brain structures, including the cingulate gyrus (CNG), thalamus (THA), and cerebellum (CB) (Fig. 4c; Supplementary Table 3), while LTG and VPA-associated unique DE genes were not enriched in any brain regions. However, the set of overlapping DE genes were enriched in multiple cerebellar structures, such as several regions of the vermis and posterior lobe and the left lateral hemisphere (Fig. 4d; Supplementary Table 4), suggesting a shared effect of all three drugs on the transcriptional regulation of cerebellar NPCs. Pathway enrichment analysis of the DE genes uniquely associated with each drug showed that Li-only genes were most strongly enriched for axon guidance processes, followed by focal adhesion, cell cycle, and RNA transport (Fig. 4e). On the other hand, the shared DE genes were enriched for pathways related to the ribosomal machinery, OXPHOS, thermogenesis, endocytosis, and the spliceosome, among others (Fig. 4e). The common association with ribosome function is in line with previous reports showing that Li inhibits the binding of transfer RNA to ribosomes [51], which results in a reversible mechanical dissociation of the ribosome into 50S and 30S subunits. Likewise, it has been shown that LTG treatment may lead to a rapid accumulation of immature 30S and 50S ribosomal subunits in bacteria [52].

DE analyses were also performed after 1 week of drug exposure (Fig. 4f–i). No DE genes were identified for Li treatment. VPA-associated (FDR < 0.1) unique [39] and shared (271) DE genes (Fig. 4g) were not enriched for any pathway or brain region. However, LTG-related unique DE genes (4381) were enriched in multiple brain stem structures (Fig. 4h; Supplementary Table 5). These LTG genes were also significantly associated with OXPHOS, thermogenesis, and autophagy pathways (Fig. 4i). With regards to the DE analyses in set 2), both 6 h and 1 week treatment resulted in the modulation of a set of DE genes that was fundamentally the same as the treatment-associated genes identified in 1), except that more genes were associated with drug exposure when both patients and CTRL were included, reflecting the increase in power due to increased sample size.

### Li-associated DE genes regulated in Li-R NPCs are enriched in OXPHOS and spliceosome pathways

**Fig. 5:**
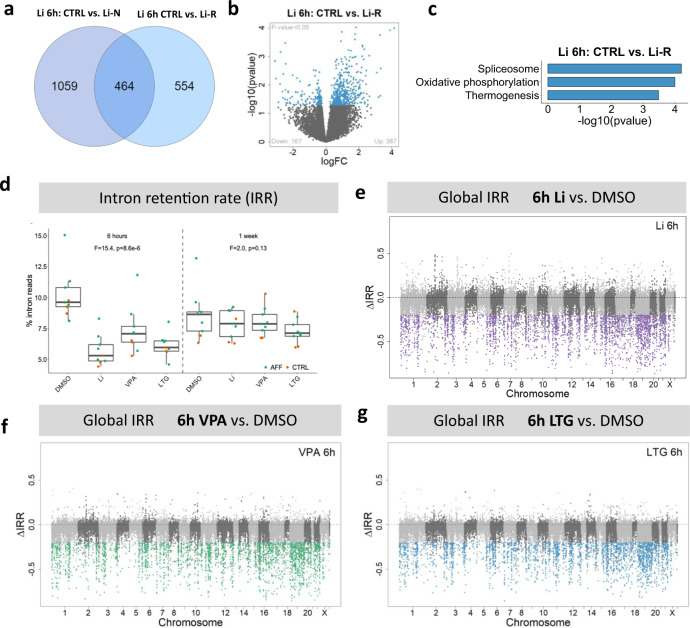
DE genes associated with Li treatment in responders show enrichment for spliceosome and OXPHOS pathways. **a** Venn diagram of DE genes associated with Li response status after Li treatment for 6 h, showing shared DE genes (464) and unique DE genes for Li-N (1059) and Li-R (554) NPCs. **b** Volcano plot of Li-R unique DE genes (*p* value < 0.05). **c** Pathway (KEGG) enrichment analysis of Li-R unique DE genes (554) showing significant enrichment for spliceosome, oxidative phosphorylation (OXPHOS) and thermogenesis pathways. **d** Initial analysis of overall intron retention rate (IRR), measured as the percentage of sequencing reads mapping to intronic regions, in NPCs after 6 h and 1 week treatment with DMSO, Li, VPA and LTG. 6 h of exposure led to significantly lower levels of intron retention for all treatments, most pronounced for Li. No comparable effect was found for 1 week exposure. F statistic and *p* value of repeated measures ANOVA tests are shown. Plots displaying transcriptome-wide per-intron IRR by chromosome location for 6 h treatment with Li (**e**), VPA (**f**) and LTG (**g**). Colored dots represent introns with significant (IR change >20% and FDR < 0.05) differences in retention rate in drug-treated samples compared to DMSO samples.

In the third set of DE analyses, we sought to identify Li-associated DE genes with differential regulation in Li-R NPCs compared to Li-N NPCs to elucidate the transcriptional mechanisms of action of Li response. After 6 h of treatment with Li, 464 genes were differentially expressed in both Li-R and Li-N groups compared to CTRL, while 1059 DE genes were unique for Li-N and 554 DE genes were unique for Li-R samples (Fig. 5a). However, these genes were only nominally significant (*p* < 0.05) and did not reach the transcriptome-wide significance threshold of FDR < 0.1. Of the 554 unique Li-R-associated genes, 387 (70%) were up-regulated and 167 (30%) were down-regulated (Fig. 5b). Interestingly, these 554 Li response-associated DE genes were enriched for spliceosome, OXPHOS, and thermogenesis pathways (Fig. 5c), where most genes annotated to the thermogenesis pathway are also involved in OXPHOS. Two of the spliceosome-associates genes were *SRSF4* and *SRSF5*, both of which were regulated by all three drugs (Supplementary Fig. 3a, b) and are essential components of the splicing machinery [53].

### Short-term treatment with mood stabilizers leads to decreased intron retention rate (IRR)

As spliceosome was one of the pathways significantly enriched in the shared treatment-associated genes and the pathway with strongest association with Li response, we investigated whether this effect was reflected in the NPC splicing activity. Although several types of alternative splicing exist, we examined the levels of intron retention rate (IRR) because it is a widespread phenomenon in mammals, affecting more than 80% of coding genes in multiple tissues [41, 54]. An initial analysis of the overall IRR, measured as the percentage of sequencing reads mapping to intronic regions, showed that 6 h of exposure to any of the drugs led to significantly lower levels of IRR, most pronounced for Li (F = 15.4, *p* = 8.6e−6) (Fig. 5d). No comparable effect for 1 week exposure was seen, although trending differences were observed that did not reach statistical significance (Fig. 5d). To investigate whether this drug-induced reduction in IRR was global or confined to specific genes or genomic regions, we used IRFinder [41] to perform an in-depth intron retention analysis. As the transcriptome-wide IRR plots show, the decrease in IRR produced by all three drugs seems to be global and not restricted to specific regions of the genome (Fig. 5e–g). We also found a significant negative correlation (*p* < 2.2e−16 for all drugs) between mean difference in IRR and mRNA expression level of each gene, meaning that the more up-regulated a transcript is in drug-treated vs. DMSO-treated samples, the fewer introns are retained. To exclude the possibility that the identified reduction in IRR was caused by DNA contamination, we used ANOVA to assess the distributions of intergenic, intronic, and exonic reads across the DMSO and drug-treated samples. Differences in intergenic proportions were not significant after correcting for intronic fractions (*F* = 0.75, *p* = 0.53; Supplementary Fig. 4d), indicating that the IRR findings were not driven by genomic DNA contamination.

### Li restores low mitochondrial oxygen consumption rate (OCR) in Li-R NPCs

Pathway enrichment analysis of the shared DE genes ((Li∩VPA∩LTG), *n* = 5558) identified OXPHOS as one of the pathways most significantly enriched (Fig. 4e). Among the most significant OXPHOS-related DE genes identified after Li treatment for 6 h were *TSPO, NDUFS7, TRADD and NDUFS8* (Supplementary Fig. 4e–h and Supplementary Table 6). OXPHOS activity is essential for the cellular bioenergetics and is required for the mitochondrial capacity to generate ATP. Therefore, we assessed the impact of Li, VPA, and LTG on mitochondrial function in the three different groups (CTRLs, Li-R and Li-N) after 6 h and 1 week of stimulation. We used the Seahorse XFe24 extracellular flux analyzer, which simultaneously measures oxygen consumption rate (OCR) and extracellular acidification rate (ECAR) from the cell supernatant, enabling a direct quantification of mitochondrial respiration and glycolysis under basal conditions and after injection of mitochondrial disruptor compounds through drug injection ports.

**Fig. 6:**
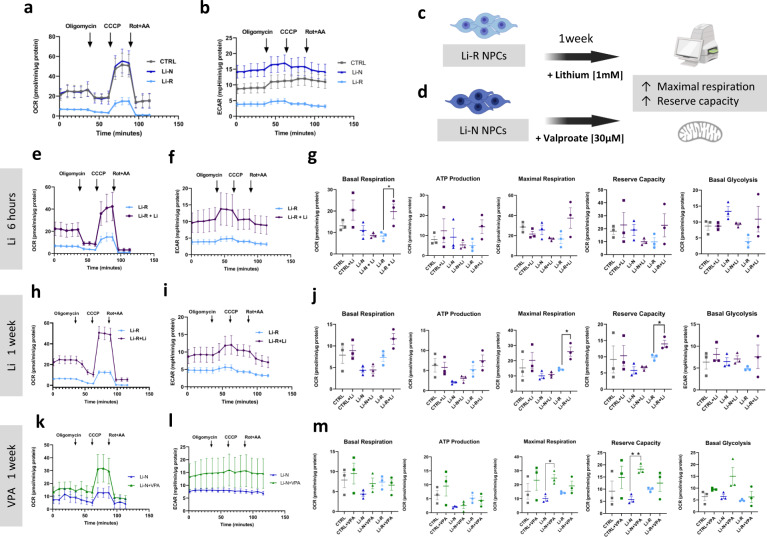
Li and VPA enhance mitochondrial OCR in patient-derived NPCs. Analysis of oxidative phosphorylation using Seahorse Analyzer. **a** Oxygen consumption rate (OCR) kinetics graph showing that Li-R patient NPCs present a tendency of decreased mitochondrial OCR, which could be indicative of dysfunctional mitochondrial function as compared to CTRL cells. **b** Extracellular acidification rate (ECAR) kinetics graph displaying glycolysis activity for all three groups. Diagram summarizing the main OCR and ECAR results after Li (**c**) and VPA (**d**) treatments. Li treatment of Li-R NPCs lead to an increase in maximal respiration and reserve capacity. VPA treatment of Li-N NPCs lead to an increase in maximal respiration and reserve capacity. **e–g** OCR (**e**) and ECAR (**f**) graphs for Li treatment for 6 h. The ECAR graph shows a tendency to increase basal glycolytic activity, but no statistically significant difference was found. **g** Basal respiration, ATP production, maximal respiration, reserve capacity and basal glycolysis after 6 h of Li treatment. Basal respiration of Li-R cells was significantly higher after Li treatment. The means of OCR parameters and basal glycolysis from untreated CTRL, Li-N and Li-R cells were compared by one-way ANOVA. The means of OCR parameters and basal glycolysis from untreated vs. treated cells for each experimental group were compared by two-tailed unpaired *t*-test. **h–j** OCR (**h**) and ECAR (**i**) graphs after Li treatment for 1 week. **j** Basal respiration, ATP production, maximal respiration, reserve capacity and basal glycolysis after 1 week Li treatment. Maximal respiration and reserve capacity of Li-R cells were significantly higher after Li treatment. **k–m** OCR (**k**) and ECAR (**l**) graphs for VPA treatment for 1 week. **m** Basal respiration, ATP production, maximal respiration, reserve capacity and basal glycolysis after 1 week VPA treatment. Maximal respiration and reserve capacity of Li-N cells were significantly higher after VPA treatment. Data was analyzed by two-tailed unpaired *t*-test. All experiments were run in quadruplicates, and values are corrected for total protein levels. Data is presented as mean ± SEM of at least two independent experiments (*n* = 3 cell lines/group). NPCs neural precursor cells, CTRL control, Li-R lithium responders, Li-N lithium non-treated, ECAR extracellular acidification rate, OCR oxygen consumption rate.

We found a tendency of decreased OCR and ECAR in NPCs from Li-R patients compared to CTRL NPCs (Fig. 6a, b), which is in line with previous studies [55]. However, the differences were not significant, which may be due to the donor variability. From the OCR and ECAR kinetics graphs, we calculated key parameters of mitochondrial respiratory function such as basal respiration, respiration linked to ATP production, maximal respiration, and reserve capacity by sequential addition of 4 mitochondria perturbing agents: oligomycin, CCCP, rotenone and antimycin. After 6 h of treatment, Li significantly increased basal respiration in Li-R NPCs (Fig. 6e, g) but not in CTRL or Li-N samples (Fig. 6g and Supplementary Fig 5a). This increased tendency was sustained after 1 week of treatment, and in addition, a significant increase in maximal respiration and reserve capacity was observed (Fig. 6h, j). No significant effect on basal glycolysis was seen in any of the conditions after Li treatment (Fig. 6f, g, i, j). With respect to VPA and LTG treatment, no effect on mitochondrial OCR and ECAR parameters were seen after 6 h (Supplementary Figs. 6a–c and 7a–f). However, 1 week of VPA exposure led to an increase in maximal respiration and reserve capacity in Li-N NPCs (Fig. 6k–m), and 1 week of treatment with LTG led to a reduction in ATP production in Li-N NPCs (Supplementary Fig. 7d–f).

### Li treatment does not rescue increased mitophagy in Li-N NPCs

A recent study found a dysregulation of mitophagy proteins in peripheral blood from BD patients [56]. Mitophagy is the selective degradation of malfunctioning mitochondria by autophagy, which often occurs to defective mitochondria and is essential for maintaining cellular viability. Therefore, we investigated whether the increase in mitochondrial respiratory parameters after Li treatment (Fig. 6e–j) was due to a reduction in mitophagy levels in the NPCs. Interestingly, Li-N NPCs presented higher mitophagy levels than both CTRL and Li-R NPCs (Supplementary Fig. 8a). However, we did not find any significant improvement after Li treatment neither after 6 h nor 1 week (Supplementary Fig. 8c).

## Discussion

One approach to gain insight into the molecular basis of affective disorders is to investigate the mechanisms of action of mood-stabilizing drugs. Recent developments in iPSC technologies have now made it feasible to study these mechanisms directly in relevant and patient-derived brain cell populations, which ensures that the disease-specific genetic background is preserved.

Here, we used transcriptional profiling and functional analyses to study the molecular effects of three commonly used mood stabilizers (Li, VPA, and LTG) in iPSC-derived NPCs from healthy controls and affective disorder patients, including both Li responders (Li-R) and Li non-treated (Li-N). Our main objective was to explore the gene expression signatures of these drugs using high-coverage RNA-seq, and to investigate the extent to which these signatures overlap and how they are related to diagnostic as well as Li response status.

### Shared gene expression signature across mood stabilizers

We observed a large number of genes and overlap of the identified DE genes associated with all three mood stabilizers (Fig. 4b, e). This is highly interesting, as three mood stabilizers with different modes of action seem to affect a specific set of genes, suggesting an expression signature with high relevance for the mood stabilizing properties of the drugs. Accordingly, we found that shared DE genes associated with all three drugs were enriched for OXPHOS, homologous recombination, biosynthesis of amino acids, protein processing in endoplasmatic reticulum, endocytosis or spliceosome pathways (Fig. 4e) among others, suggesting that these shared pathways and molecular mechanisms involved in the correct functioning of the cell may underlie some of the essential mood-stabilizing properties of these drugs. In order to explore a potential common functional outcome derived from this shared expression profile, we further investigated two of these significant pathways, OXPHOS and spliceosome, identifying relevant functional effects of the mood stabilizers by regulating intronic retention (Fig. 5) and mitochondrial properties (Fig. 6).

### Short-term reduction in IRR after mood stabilizers treatment

Recent genome-wide association studies in BD, MDD and schizophrenia have shown that most of the associated risk variants are located in non-coding regions of the genome [57]. Since these regions harbor many regulatory elements, transcriptional regulatory mechanisms are likely to play a significant role in mental disorders. Moreover, evidence from post-mortem brain tissue studies suggests an increased rate of dysfunctional splicing in brain samples from individuals with psychiatric disorders [58]. For instance, disruptions in the splicing machinery caused specifically by increased *SRSF3* mRNA expression have been associated with the pathophysiology of BD [59].

Pre-mRNA splicing and alternative splicing are key mechanisms by which the proteomic diversity of eukaryotic genomes is expanded. In fact, mutations in pre-mRNA molecules have been linked to splicing dysfunction that give rise to over 200 human diseases [60]. Intron retention (IR) is the least studied of all types of alternative splicing, likely because these splicing events were thought to be mere consequences of mis-splicing. In plants and unicellular eukaryotes, IR is the most common form of alternative splicing, and it is also a common phenomenon in mammals [54]. Here, we show that short-term (6 h) exposure to Li, VPA, and LTG leads to a substantial decrease in intron retention rate (IRR) (Fig. 5d). This initial impact on IRR seems to be an immediate effect of drug exposure as it did not last after 1 week of treatment. Moreover, our findings indicate that the short-term reduction in IRR is transcriptome-wide rather restricted to specific genes or chromosomal regions (Fig. 5e–g). It was generally assumed that mis-splicing, leading to the retention of introns, would have no physiological consequences other than reducing gene expression by nonsense-mediated decay. Yet, recent discoveries have highlighted the pivotal role that IR plays in human biology [61, 62], emerging thus as an unexpected generator of transcriptomic diversity in various stages of cell differentiation in mammals [61, 62]. Therefore, even though we did not find a long-lasting effect on IRR after 1 week, an initial boosting effect after drug treatment might potentially be enough to induce long-lasting consequences in the treated cells.

Interestingly, we found that *SRSF4* and *SRSF5*, encoding two members of the splicing factor family, were regulated after treatment with all three drugs (Supplementary Fig. 3a, b). *SRSF5* is of particular interest as it is ubiquitously expressed in mammals [53]. Thus, our findings indicate that the mechanisms of action of Li, VPA, and LTG in regulating mood stabilization may partly stem from the regulation of the spliceosome and its associated genes and consequently of IRR regulation.

### Li improves mitochondrial oxygen consumption rate (OCR)

Multiple lines of evidence suggest a possible involvement of mitochondrial deficits in major psychiatric disorders [10, 63] and current stem cell technology is dramatically improving our understanding of the bioenergetics alterations in these diseases [10, 25]. Accordingly, we found that the identified DE genes associated with all three drugs (Fig. 4e), as well as the genes associated with Li response, were most strongly enriched for the mitochondrial OXPHOS pathway (Fig. 5c). Mitochondria play a crucial role in neuronal functions, especially in energy production, calcium signaling, and the generation of reactive oxygen species. Although the molecular effects of mood stabilizers on mitochondria are still not completely understood, several studies have unraveled potential mechanisms. For example, Li and VPA have been reported to enhance mitochondrial function and protect against methamphetamine-induced mitochondrial toxicity [14, 15], in line with our results of enhanced OCR parameters (Fig. 6). Li treatment has also been found to increase lifespan and mitochondrial energy in *C. elegans* [64]. However, a recent study found no positive effect on an abnormally low mitochondrial membrane potential after Li treatment of iPSC-derived neurons from BD patients [10]. Apart from VPA and Li, LTG has also been found to have beneficial effects on mitochondrial toxicity in mitochondrial epilepsy [65].

Mammalian cells generate ATP by mitochondrial oxidative phosphorylation and non-mitochondrial glycolytic metabolism. Using the Seahorse XFe24 extracellular flux analyzer, we assessed the cellular bioenergetics of drug-treated NPCs. We found that 6 h of Li treatment led to a significant increase in basal respiration, and 1 week treatment with Li resulted in a significant increase in maximal respiration and reserve capacity only in Li-R NPCs (Fig. 6g, j). These data suggest that Li triggers an immediate increase in mitochondrial respiration capacity, and that this effect is specific to Li responders and stable over time. Furthermore, we found an increase in maximal respiration and reserve capacity in Li-N NPCs after 1 week treatment with VPA (Fig. 6k–m), in line with a previous study where VPA increased basal respiration in neural progenitors NT2-N cells [66]. This increase in mitochondrial respiration parameters after 1 week of treatment may explain why VPA has become the main adjunctive treatment to Li in the treatment of mania in BD patients [4]. In contrast, 1 week treatment with LTG led to a decrease in ATP production (Supplementary Fig. 7), which is contrary to a previous study where LTG increased basal respiration in NT2-N cells [66]. These apparent discrepancies may be due to the fact that a different cell type and drug concentration was used in our study.

The mitochondrial electron transport chain (ETC) is a key parameter used to evaluate mitochondrial function. It has been reported that Li treatment in BD patients increases the ETC complex I activity in vivo during depressive episodes [67]. In line with this, we found that Li had an up-regulating effect on the *NDUFS7* gene (Supplementary Fig. 8), which encodes a highly conserved subunit of the mitochondrial complex I, involved in proton translocation [68]. Likewise, we found an increase in *TSPO* after Li treatment (Supplementary Fig. 8a), which has also been associated with complex I activity [69] and might explain the observed increased tendency in ATP production and other mitochondrial parameters (Fig. 6g–j). Interestingly, previous studies have reported a dysregulation of *TSPO* in peripheral blood from BD patients [56] and low *NDUFS7* in BD post-mortem tissue [70], suggestive of defective ETC complex I. Taken together, these results suggest a key mechanistic role of Li in mitochondrial bioenergetics. In particular, our results support that Li enhances OCR parameters in Li-R patients, probably through the regulation of mitochondrial complex I components.

### Mood stabilizers affect genes enriched in cerebellum

Even though the cerebellum only accounts for 10% of the total brain volume, it contains 80% of all neurons [71]. Historically, cerebellar regions have received little attention in pathological and imaging studies on affective disorders like MDD and BD. However, in the last two decades there has been a growing interest in its possible role in mood disorders [72]. An increasing body of literature has changed the view of the cerebellum from a structure implicated uniquely in motor function to a region involved in cognition and emotion as well [73]. For example, imaging studies have demonstrated the existence of anatomical links between several cerebellar regions and the sensorimotor and association areas of the brain cortex [74]. Moreover, there is increasing evidence that these cerebellum connections with cortical areas are involved in the pathophysiology of psychiatric disorders [72]. Accordingly, we found that Li, VPA, and LTG-associated shared DE genes were enriched in cerebellar regions (Fig. 4d and Supplementary Table 4), suggesting that the beneficial effect of these three drugs might partly occur in the cerebellum, which has previously also been linked to neurological side effects of Li. Apart from the cerebellum, the cortex, amygdala and hippocampus are the brain regions that are primarily affected in BD [75]. The fact that not many cortical regions were enriched in the treatment-associated DE genes we identified (Fig. 4) might be explained by the fact that NPCs are mainly found in ventricular-subventricular regions, which we found as enriched regions instead.

## Limitations of the study

This study has two main limitations: the small number of participants, and the sub-optimal clinical phenotype of patient’s responsiveness to Li, which need to be considered when interpreting the results. Although definitions of outcome vary between studies, responsiveness is typically determined by comparing recurrence rate, duration and severity of mood episodes before and during lithium treatment. However, due to the cross-sectional design we were not able to assess changes of illness activity. Thus current symptom level during treatment was applied to indicate differences in responsiveness.

In summary, our study revealed widespread transcriptional effects of Li, VPA, and LTG in iPSC-derived NPCs with potential involvement in clinical drug response. While some of these effects were unique to each drug, a substantial overlap in the drugs’ gene expression signatures was observed, suggesting that shared pathways and molecular mechanisms may underlie the mood-stabilizing properties of these drugs. The findings provide further support for a key role of mitochondrial regulation in the molecular mechanisms of mood stabilizers, and suggest novel mechanisms related to the splicing machinery and intron retention, which may be utilized as potential targets of new therapeutic agents.

## Supplementary information

Supplementary figure legends

Supplementary Tables

Supplementary Figure 1

Supplementary Figure 2

Supplementary Figure 3

Supplementary Figure 4

Supplementary Figure 5

Supplementary Figure 6

Supplementary Figure 7

Supplementary Figure 8
