# Supplementary material for: Lithium increases mitochondrial respiration in iPSC-derived neural precursor cells from lithium responders
Source: Mol Psychiatry. 2021 Jun 1;26(11):6789–805. doi: 10.1038/s41380-021-01164-4 (PMC8760072; doi:10.1038/s41380-021-01164-4)
Supplement: Supplementary file 1 — Supplementary figure legends [file 41380_2021_1164_MOESM1_ESM.docx]

**SUPPLEMENTARY FIGURE LEGENDS**

**Supplementary Figure 1.**

**(A)** IPSC immunofluorescence staining for the rest of the lines not shown in Figure 2. Nuclear Oct4 and Nanog pluripotency markers are shown in the merged image. Cell nuclei were stained with DAPI. **(B)** Alkaline phosphatase (AP) staining showing positive expression in iPSC colonies from each line used in the study not shown in Figure 2. Scale bars 100 µm. **(C)** Karyotype for the rest of the lines not shown in Figure 2, assessed by KaryoStat™ analysis. Somatic and sex chromosomes are displayed together. The y-axis displays signal intensities in log2-space for microarray probes. A copy number (CN) value of 2 represents a normal copy number state. Chromosomal gains are represented by a value of 3. Chromosomal losses are represented by a value of 1. Pink, green and yellow colours indicate each individual chromosome probe´s raw signal. The blue line represents the normalized probe signal used to identify copy number and aberrations. The same chromosomal loss of ~7000 kb was detected in two of the patient lines (#5 and #6) at the 6q24.3 locus (partial chromosomal loss).

**Supplementary Figure 2.**

**(A)** Normalized and standardized expression of genes in the deleted region for patient donors #5 and #6 (chromosome 6q24.3, ~7MB). As shown in the plot, most of these genes were either not expressed or lowly expressed in the two samples with the deletion. These genes were excluded before DE analyses. Pseudogenes as well as some genes not surviving pre-filtering were not included in the plot. **(B)** NPCs immunofluorescence staining of Nestin, Sox1 and Sox2 for the rest of the lines not shown in Figure 2. Scale bars 100 µm. NPC: neural precursor cells; IPSC: induced pluripotent stem cells; CTRL: control; Li-R: lithium responders; Li-N: lithium non-treated. **(C)** PCA analysis plots of all 73 samples by donor and treatment duration based on the 1000 most variant genes. The samples show tendency of clustering on diagnosis (CTRL vs. AFF). This is especially apparent when clustering across PCs 3 and 4, explaining a relatively small portion of the total variation.

**Supplementary Figure 3.**

**(A-B)** Gene expression levels of splicing factor 4 (*SRSF4*) **(A)** and splicing factor 5 (*SRSF5*) **(B)** genes after 6 hours and 1 week treatment with DMSO, Li, VPA and LTG. Both genes displayed significant differences in expression across the different treatment conditions.

**Supplementary Figure 4.**

Scatter plots showing correlations between mean changes in IRR rates (treatment vs. DMSO) and expression levels for each gene. A significant negative correlation was found for 6 hours Li **(A)**, VPA **(B)** and LTG **(C)** treatment, meaning that the more expressed a transcript is in drug-treated cells compared to DMSO cells, the fewer introns tend to be retained. **(D)** Genomic origin plot showing percentage of reads stemming from intergenic, intronic, and exonic regions after 6 hours treatment. Differences in intergenic proportions were not significant after correcting for intronic fractions, indicating that the IRR results were not driven by DNA contamination. F statistic and p-value of repeated measures ANOVA tests are shown. Normalized gene expression of the mitochondrial genes *TSPO* **(E)**, *NDUFS7* **(F)**, TRADD **(G)** and *NDUFS8* **(H)**, which were the most significant DE genes related to the OXPHOS pathway.

**Supplementary Figure 5.**

**(A, C)** Oxygen consumption rate (OCR) and **(B, D)** Extracellular acidification (ECAR) kinetics graphs for CTRL, Li-N and Li-R groups treated with Li for 6 hours and 1 week. All experiments were run in quadruplicates, and all values are normalized to total protein levels. Data is presented as mean ± SEM. (n = 3 cell lines/group). NPCs: neural precursor cells; CTRL: control; Li-R: lithium responders; Li-N: lithium non-treated; ECAR: extracellular acidification rate; OCR: oxygen consumption rate.

**Supplementary Figure 6.**

**(A, D)** Oxygen consumption rate (OCR) and **(B, E)** Extracellular acidification (ECAR) kinetics graphs for CTRL, Li-N and Li-R groups treated with VPA for 6 hours and 1 week. **(C)** Basal respiration, ATP production, maximal respiration, reserve capacity and basal glycolysis mitochondrial parameters for each group and drug duration are given. All experiments were run in quadruplicates, and all values were normalized to total protein levels in the well. Data is presented as mean ± SEM of at least two independent experiments (n = 3 cell lines/group).

The means of OCR parameters and basal glycolysis from untreated vs. treated cells for each experimental group were compared by two-tailed unpaired t-test. NPCs: neural precursor cells; CTRL: control; Li-R: lithium responders; Li-N: lithium non-treated; ECAR: extracellular acidification rate; OCR: oxygen consumption rate.

**Supplementary Figure 7.**

**(A, D)** Oxygen consumption rate (OCR) and **(B, E)** Extracellular acidification (ECAR) kinetics graph for CTRL, Li-N and Li-R groups treated with LTG for 6 hours and 1 week. **(C, F)** Basal respiration, ATP production, maximal respiration, reserve capacity and basal glycolysis mitochondrial parameters for each group and drug duration are given. **(G)** Diagram summarizing the main Seahorse results after LTG treatment. LTG treatment of patient Li-N NPCs lead to a decrease in mitochondrial respiration linked to ATP production. All experiments were run in quadruplicates, and all values were normalized to total protein levels in the well. Data is presented as mean ± SEM of at least two independent experiments (n = 3 cell lines/group). The means of OCR parameters and basal glycolysis from untreated vs. treated cells for each experimental group were compared by two-tailed unpaired t-test. NPCs: neural precursor cells; CTRL: control; Li-R: lithium responders; Li-N: lithium non-treated; ECAR: extracellular acidification rate; OCR: oxygen consumption rate.

**Supplementary Figure 8.**

**(A)** Graph showing the mitophagy dye fluorescence intensity average for CTRL, Li-N and Li-R NPCs analyzed by flow cytometry. Significantly higher mitochondrial mitophagy levels were found in Li-N NPCs (two-tailed unpaired t-test). **(B)** Representative histograms from flow cytometry analysis showing increased mitophagy in Li-N NPCs in comparison with CTRL and Li-R cells. **(C)** Treatment with Li 1mM and 10mM had no effect in any of the groups. Data were analysed by two-tailed unpaired t-tests, and are presented as mean ± SD.
