# Supplementary figures and images for: Lithium increases mitochondrial respiration in iPSC-derived neural precursor cells from lithium responders
Source: Mol Psychiatry. 2021 Jun 1;26(11):6789–805. doi: 10.1038/s41380-021-01164-4 (PMC8760072; doi:10.1038/s41380-021-01164-4)

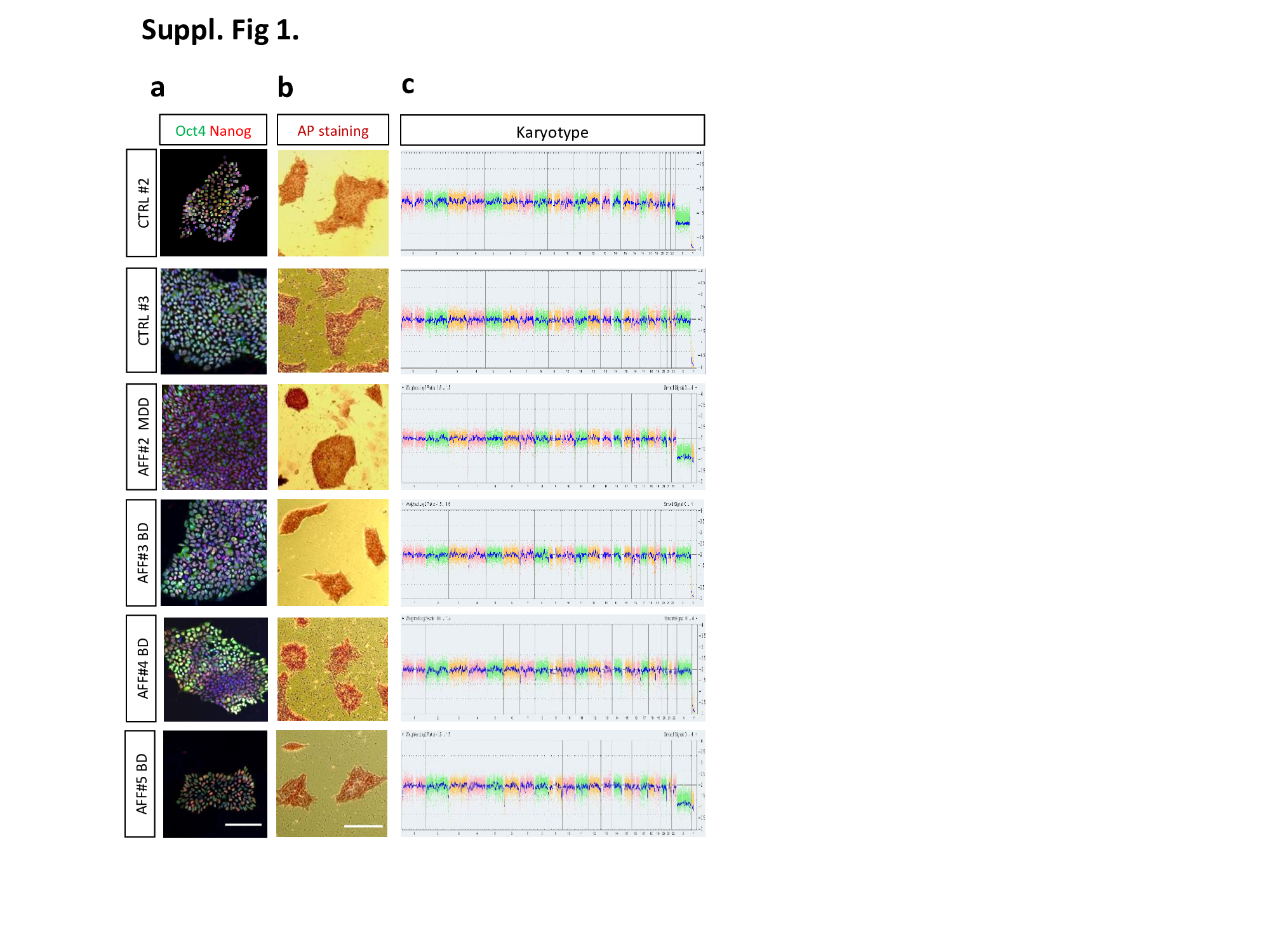

Supplement: Supplementary file 3 — Supplementary Figure 1 [file 41380_2021_1164_MOESM3_ESM.tif]

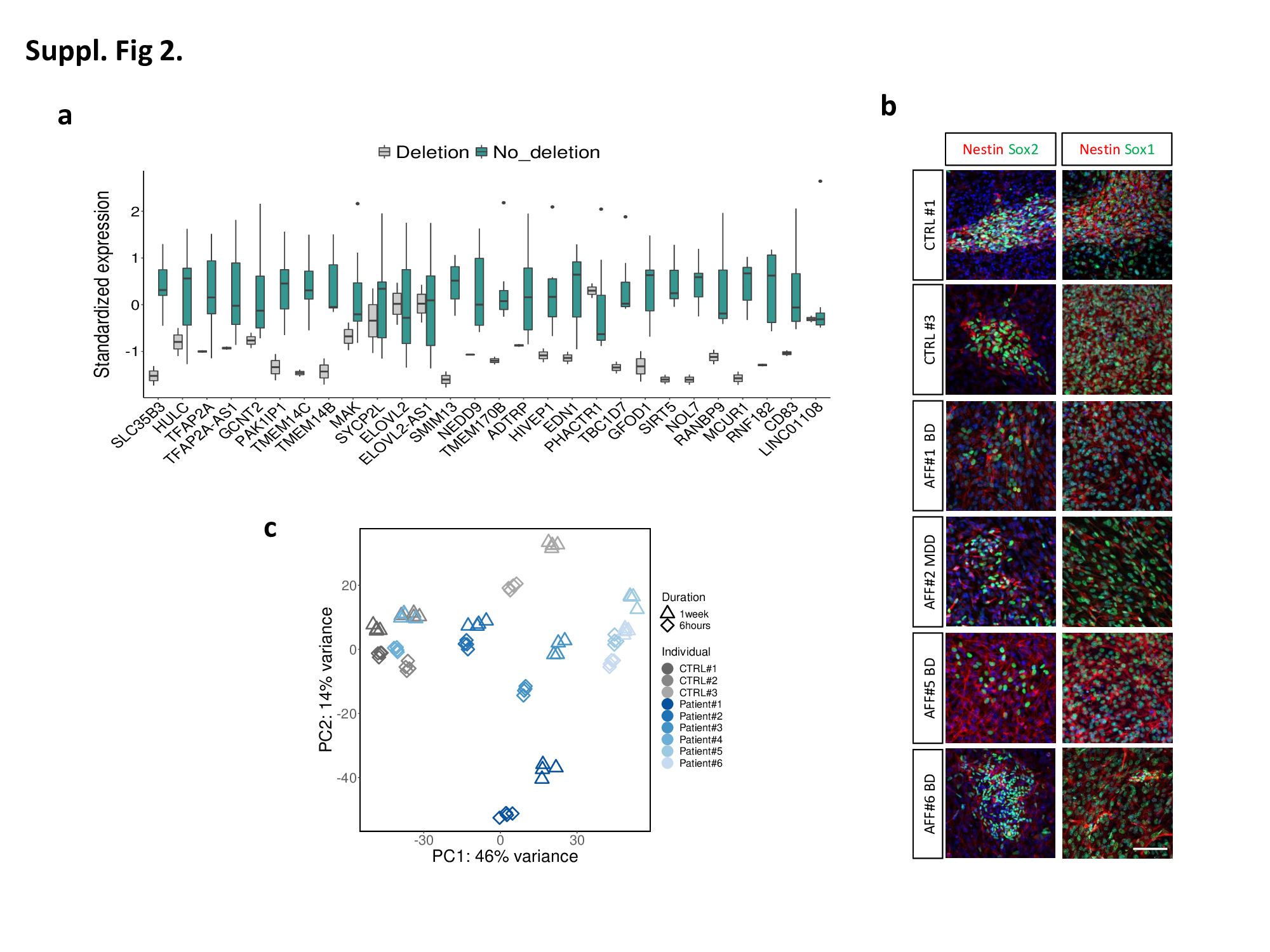

Supplement: Supplementary file 4 — Supplementary Figure 2 [file 41380_2021_1164_MOESM4_ESM.tif]

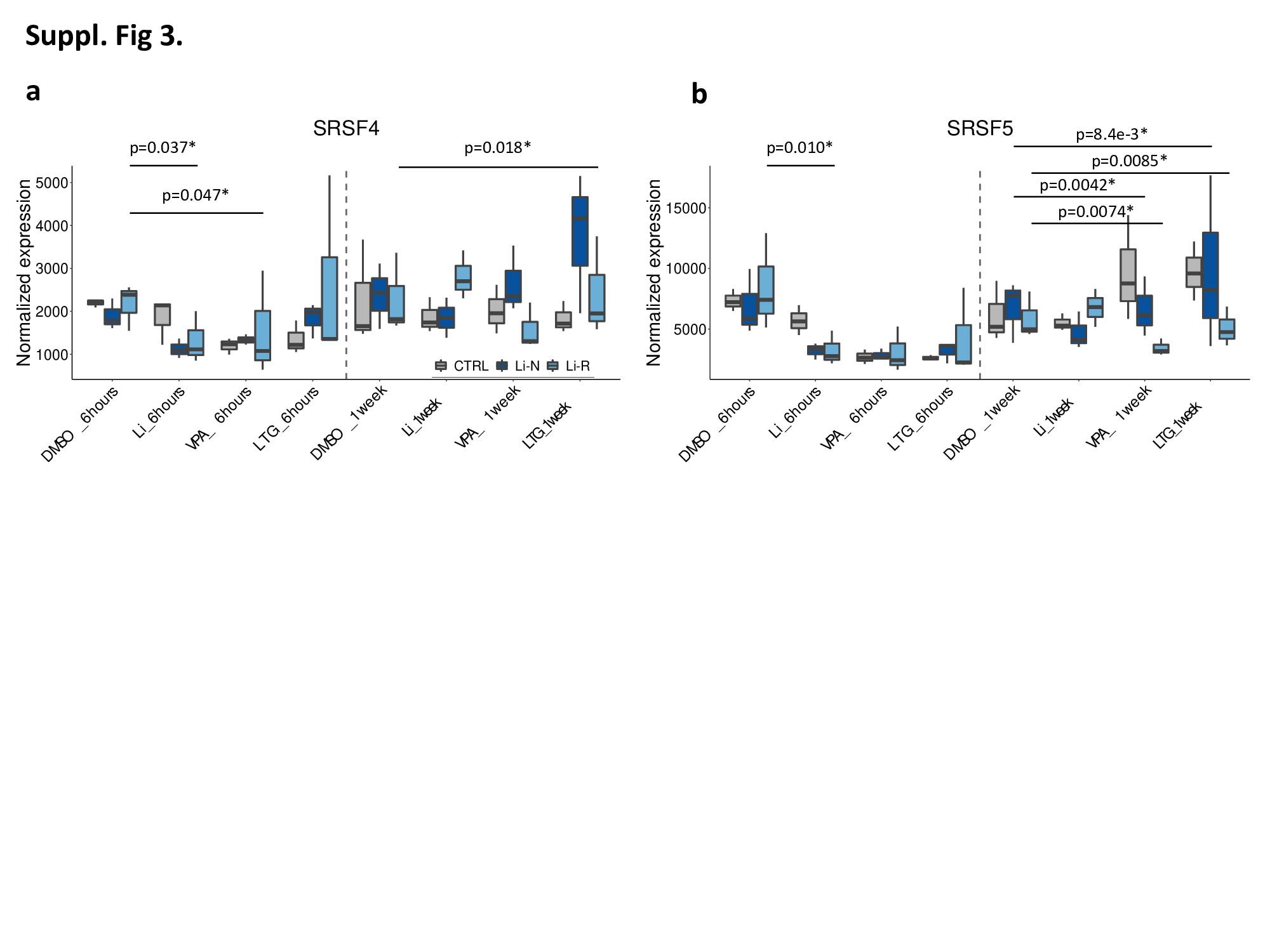

Supplement: Supplementary file 5 — Supplementary Figure 3 [file 41380_2021_1164_MOESM5_ESM.tif]

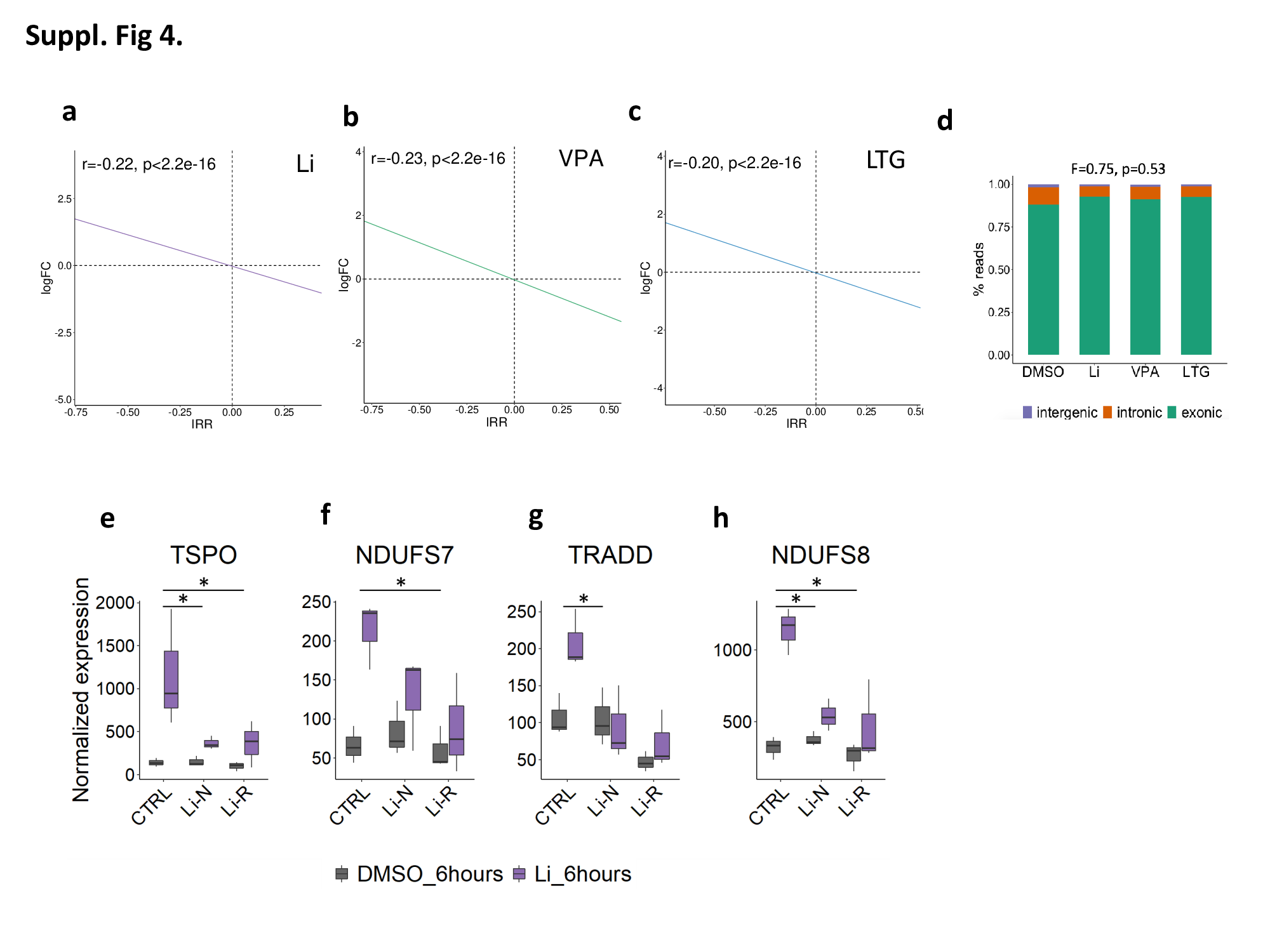

Supplement: Supplementary file 6 — Supplementary Figure 4 [file 41380_2021_1164_MOESM6_ESM.tif]

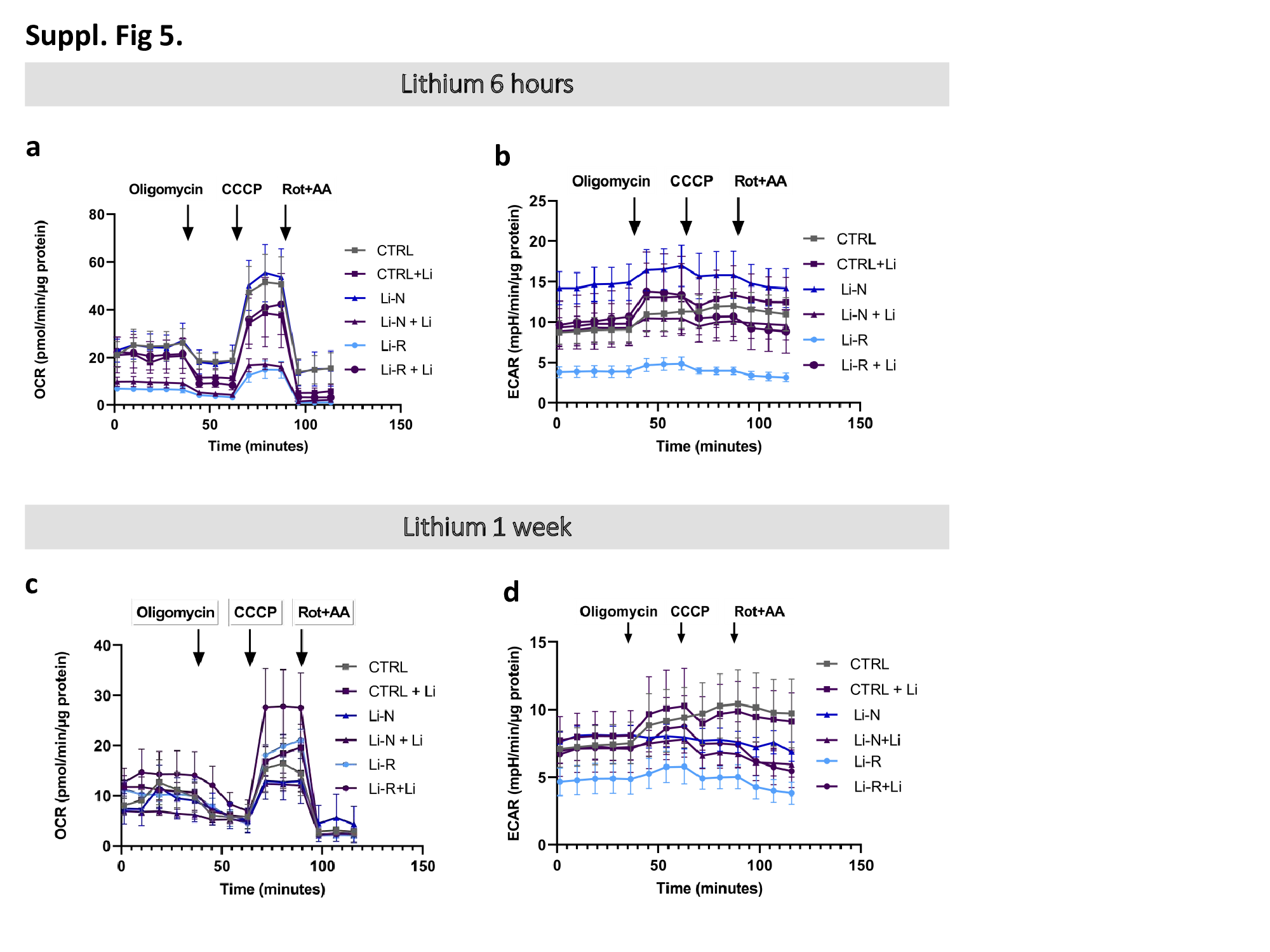

Supplement: Supplementary file 7 — Supplementary Figure 5 [file 41380_2021_1164_MOESM7_ESM.tif]

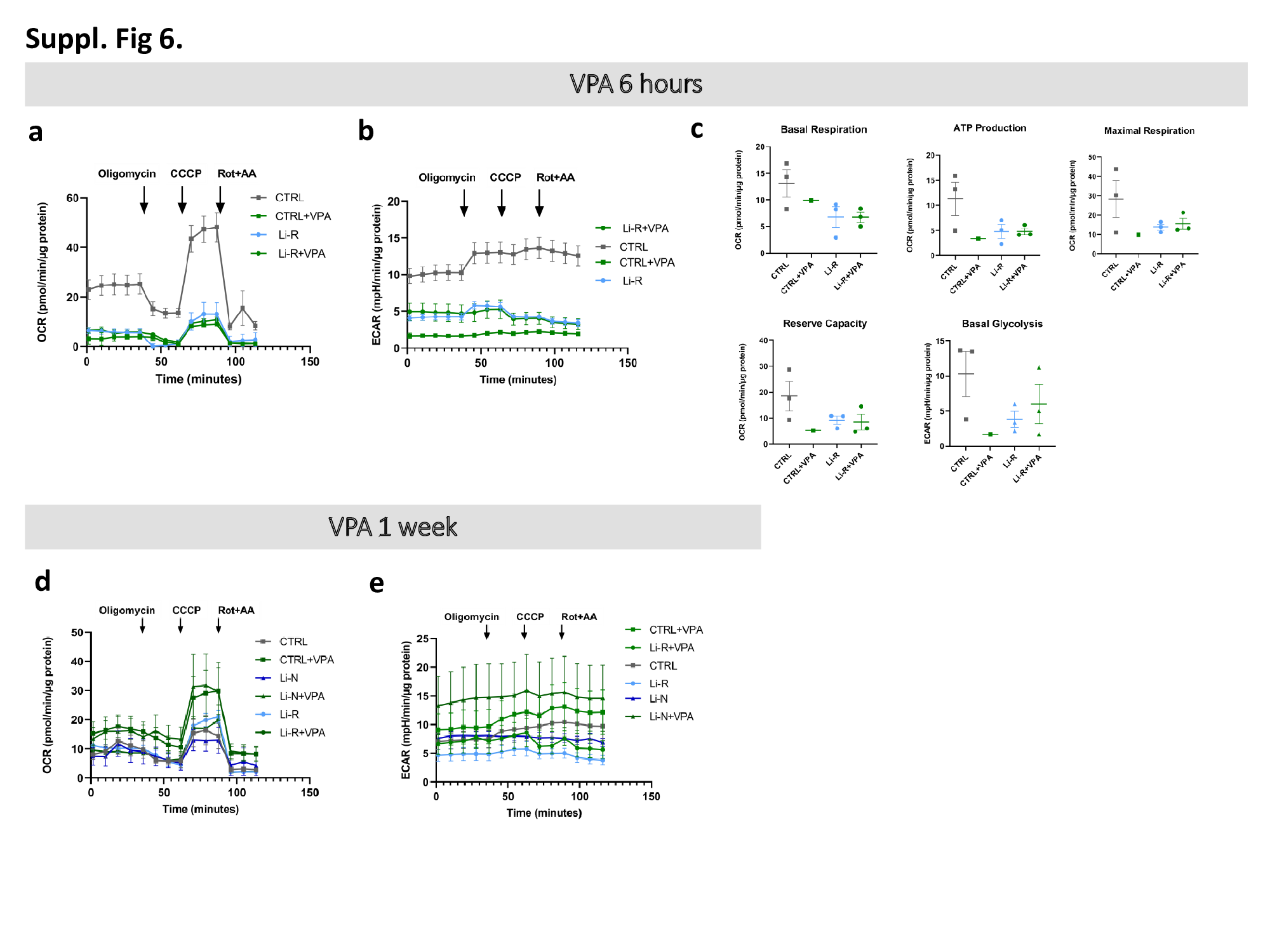

Supplement: Supplementary file 8 — Supplementary Figure 6 [file 41380_2021_1164_MOESM8_ESM.tif]

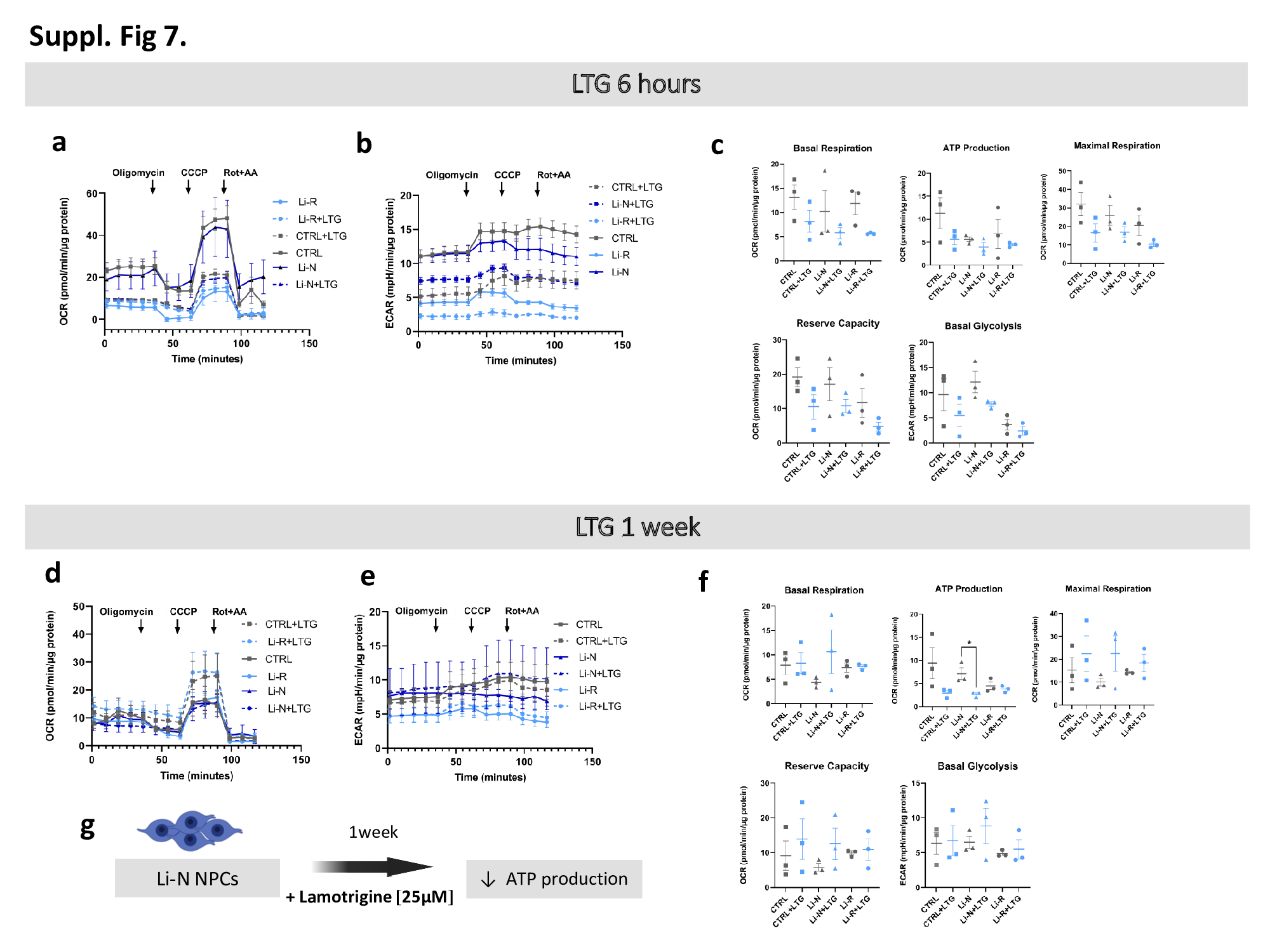

Supplement: Supplementary file 9 — Supplementary Figure 7 [file 41380_2021_1164_MOESM9_ESM.tif]

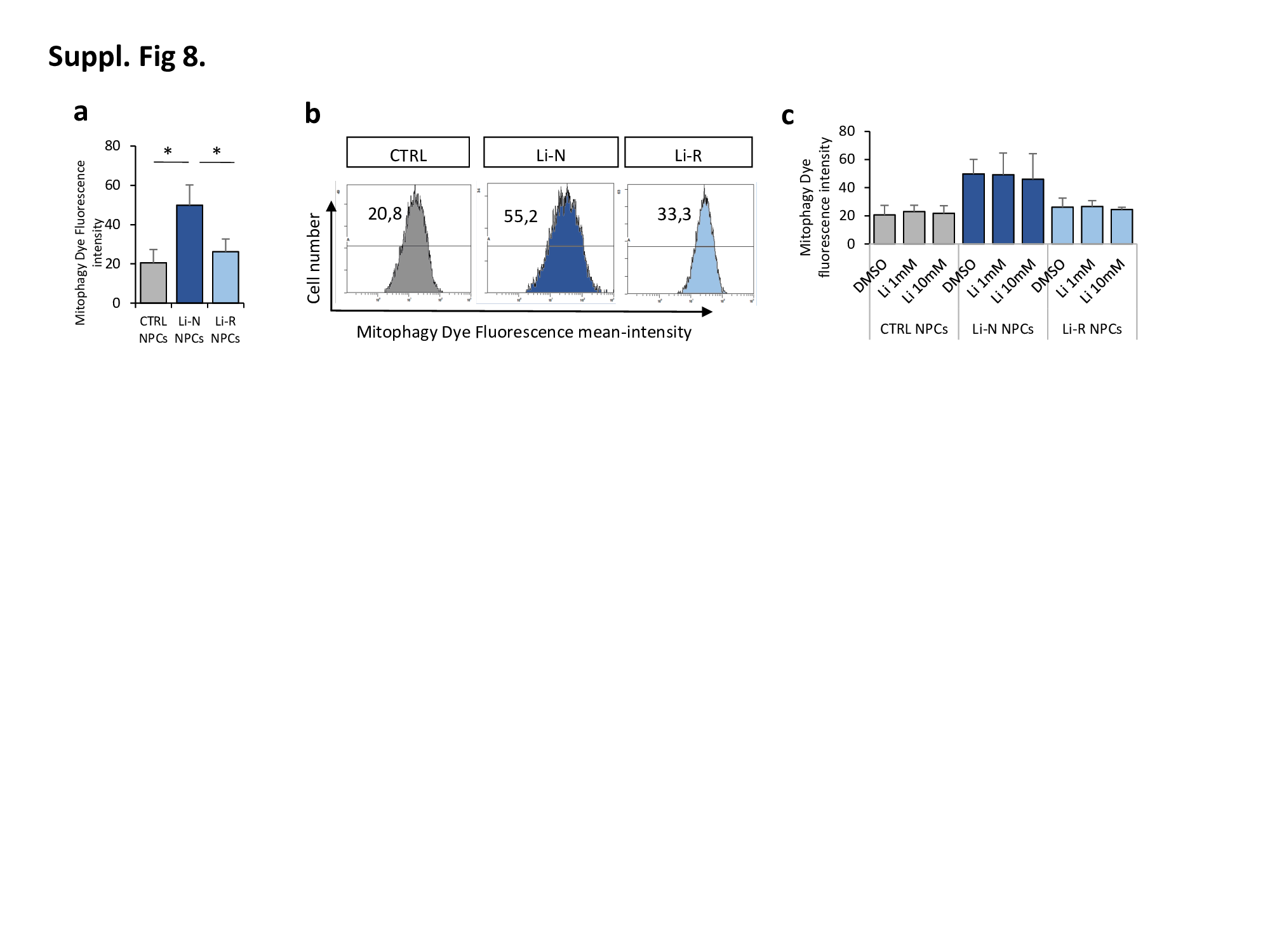

Supplement: Supplementary file 10 — Supplementary Figure 8 [file 41380_2021_1164_MOESM10_ESM.tif]
